# Editorial: Recent advances in progestin-primed ovarian stimulation

**DOI:** 10.3389/fendo.2022.1004352

**Published:** 2022-11-04

**Authors:** Qiuju Chen

**Affiliations:** Shanghai Ninth People’s Hospital, School of Medicine, Shanghai Jiao Tong University, Shanghai, China

**Keywords:** In vitro fertilization, controlled ovarian stimulation, progestin-primed ovarian stimulation (PPOS), poor ovarian response (POR), GnRH analogue

Controlling premature ovulation is a key challenge in assisted reproduction. Gonadotropin-releasing hormone (GnRH) analogues are widely used to suppress pituitary activity, and their efficacy and safety have been confirmed over forty years of use (1, 2). Recently, more flexible protocols have been proposed with the aid of vitrification cryopreservation; for example, progestin has been extensively used for the prevention of premature ovulation as a substitute for GnRH analogue. This progestin-primed ovarian stimulation (PPOS) has been accepted as an important regimen for ovarian stimulation (3, 4).

However, the potential of PPOS in clinical practice remains to be determined. In this Research Topic, the efficacy, safety, and potential application scope of PPOS are discussed, especially in relation to certain demographics such as women with advanced reproductive age, poor ovarian response, and endometrioma.

Two trials described the pregnancy outcomes of PPOS in the poor prognosis and common protocols of mild stimulation GnRH antagonists were used as the control. The primary endpoints were cumulative live birth rate. A retrospective trial of 730 women with advanced reproductive age and diminished ovarian reserve (DOR) (139 PPOS and 600 mild stimulation) showed that PPOS obtained more oocytes/embryos, comparable reproductive outcomes, and better control of premature luteinizing hormone (LH) surge than mild stimulation (Tu et al.). In another trial, a total of 1,329 women who met the Patient-Oriented Strategies Encompassing IndividualizeD Oocyte Number (POSEIDON) criteria were collected, and the cumulative birth rate of PPOS was comparable to that of GnRH antagonist protocols. In the POSEIDON group 1 population, the GnRH antagonist protocols resulted in a shorter time to live birth (Du et al.).

Although the exact pathophysiology of endometrioma in infertility remains under discussion, a retrospective trial of patients with endometrioma was used to compare PPOS, ultra-long GnRHa, and GnRHan protocol. PPOS showed inferior reproductive outcomes compared to ultra-long protocol in terms of clinical pregnancy and live birth. However, no significant difference was found in clinical pregnancy and live birth between PPOS protocol and GnRH antagonist protocol (Yang et al.).

To optimize the PPOS regimen, two kinds of progestins (dydrogesterone and medroxyprogesterone) were compared in women with poor ovarian response; the results demonstrated that both combinations were useful options for PPOS protocols (Zhang et al.). In addition, progestins were started simultaneously with gonadotrophins (fixed PPOS) or later in the cycle depending on follicle growth (flexible PPOS). Flexible and fixed PPOS regimens had no appreciable differences regarding mature oocyte yield and the incidence of premature LH surge (5). Although it is limited by the small sample, the trial opened a new possibility to modulate the PPOS regimen.

The findings of the meta-analysis show potential for the consequences of low risk ovarian hyperstimulation syndrome (OHSS), with good controllability for LH surge and comparable pregnancy outcomes to GnRH analogue (Guan et al.).

Overall, PPOS provides an attractive alternative, especially for refractory cases such as advanced reproductive age and low ovarian reserve, therefore PPOS is an irreplaceable protocol in controlled ovarian stimulation.

## Author contributions

The study was funded by the Cross-disciplinary Research Fund of Shanghai Ninth People's Hospital, Shanghai Jiaotong University School of Medicine (2020-014).

## Conflict of interest

The author declares that the research was conducted in the absence of any commercial or financial relationships that could be construed as a potential conflict of interest.

## Publisher’s note

All claims expressed in this article are solely those of the authors and do not necessarily represent those of their affiliated organizations, or those of the publisher, the editors and the reviewers. Any product that may be evaluated in this article, or claim that may be made by its manufacturer, is not guaranteed or endorsed by the publisher.
